# Cep70 and Cep131 contribute to ciliogenesis in zebrafish embryos

**DOI:** 10.1186/1471-2121-10-17

**Published:** 2009-03-02

**Authors:** Christopher J Wilkinson, Matthias Carl, William A Harris

**Affiliations:** 1 Department of Physiology, Development and Neuroscience, University of Cambridge, Downing Street, Cambridge, CB2 3DY, UK; 2 Department of Anatomy and Developmental Biology, University College London, Gower Street, London, WC1E 6BT, UK; 3 Present address : School of Biological Sciences, Royal Holloway, University of London, Egham, Surrey, TW20 0EX, UK; 4 Present address : University of Heidelberg, Department of Cell and Molecular Biology, Faculty of Medicine Mannheim, 68167 Mannheim, Germany

## Abstract

**Background:**

The centrosome is the cell's microtubule organising centre, an organelle with important roles in cell division, migration and polarity. However, cells can divide and flies can, for a large part of development, develop without them. Many centrosome proteins have been identified but the roles of most are still poorly understood. The centrioles of the centrosome are similar to the basal bodies of cilia, hair-like extensions of many cells that have important roles in cell signalling and development. In a number of human diseases, such Bardet-Biedl syndrome, centrosome/cilium proteins are mutated, leading to polycystic kidney disease, situs inversus, and neurological problems, amongst other symptoms.

**Results:**

We describe zebrafish (Danio rerio) embryos depleted for two uncharacterised, centrosome proteins, Cep70 and Cep131. The phenotype of these embryos resembles that of zebrafish mutants for intraflagellar transport proteins (IFTs), with kidney and ear development affected and left-right asymmetry randomised. These organs and processes are those affected in Bardet-Biedl syndrome and other similar diseases. Like these diseases, the root cause of the phenotype lies, in fact, in dysfunctional cilia, which are shortened but not eliminated in several tissues in the morphants. Centrosomes and basal bodies, on the other hand, are present. Both Cep70 and Cep131 possess a putative HDAC (histone deacetylase) interacting domain. However, we could not detect in yeast two-hybrid assays any interaction with the deacetylase that controls cilium length, HDAC6, or any of the IFTs that we tested.

**Conclusion:**

Cep70 and Cep131 contribute to ciliogenesis in many tissues in the zebrafish embryo: cilia are made in cep70 and cep131 morphant zebrafish embryos but are shortened. We propose that the role of these centrosomal/basal body proteins is in making the cilium and that they are involved in determination of the length of the axoneme.

## Background

The centrosome is an approximately one micrometre-cubed organelle that acts as the microtubule organising centre in higher eukaryotic cells [1,2]. It consists of two cylindrical centrioles, built from microtubules, surrounded by a protein matrix. During cell division, the centrosome is duplicated contemporaneously with DNA and the two centrosomes contribute to the formation of the poles of the mitotic spindle that segregate the duplicated chromosomes faithfully between daughter cells [3]. For almost a century, the centrosome was seen as an essential component of the cell cycle, especially mitosis [4]: frog eggs with no centrosomes do not divide [5]; sea urchin eggs with too many undergo multipolar divisions [6].

The requirement of centrosomes for cell division has been severely tested over the last few years. In cell culture, the centrosome can be removed or obliterated with a laser and the cell will still divide [7,8]. In Drosophila, mitotic centrosomes are not necessary for the development of the centrosomin mutant [9]. DSas-4 mutant flies can even develop to maturity in the absence of centrosomes [10], although they rely on maternal stores of protein for early embryogenesis [11] and they die soon after hatching by drowning in their food or from dehydration [10]. This raises the question of what is the precise role of the metazoan centrosome and what all the hundred or so proteins in the complex do. Part of the answer might lie in the other cellular structure that is formed from centrioles.

Basal bodies are centriole-like structures observed underneath the cell membrane at the base of cilia, hair-like extensions of the cell membrane [1,12]. These cilia range from the highly motile, such as those that line the trachea, to the immotile and highly specialised, such as the connecting cilium to the outer segments of photoreceptors [13]. Virtually all vertebrate cells have a cilium [12,14,15], though for many cell types the seemingly inactive primary cilium has the appearance of a relic organelle [16]. The basal bodies can be made from the pre-existing centrosomal centrioles that migrate to the surface, with duplication in multi-ciliated cells, or de novo [16,17].

Research into the cilium has undergone a resurgence recently with their linkage to a number of inherited human diseases and the discovery of their role in a number of important developmental processes [12,18]. Primary cilia dyskinesia (PCD aka immotile ciliary syndrome) was shown to be due to abnormal cilia in the mid-seventies [19]. More recently, polycystic kidney disease (PKD) has been linked to the condition of cilia in the kidney tubules [20,21]. Cilia in the node, a fluid-filled compartment also known as Kupffer's Vesicle in zebrafish [22,23], are involved in initiation of left-right asymmetry [24,25] This explains the situs inversus often associated with PCD [26] and observed in a targeted mouse mutant for the gene, Tg737, which encodes the Polaris protein [27]. This mutant, an allele of the Tg737^orpk ^hypomorph which models PKD [28], affects the mouse homologue of the Chlamydomonas IFT88 protein [20], one of the family of intraflagellar transport proteins (reviewed in [29]) that move other proteins up and down the cilium in conjunction with motor proteins.

Bardet-Biedl, Alstrom and Oral-facial-digital syndromes are a number of other human diseases described whose pleiotropic and overlapping symptoms include polycystic kidney disease, situs inversus, retinal dystrophy and neurological problems [30-33]. The products of the genes mutated in these diseases localise to centrosomes and basal bodies of cilia [30,33,34].

A large portion of the centrosome inventory consists of novel and uncharacterised proteins often with no domains to give clues as to their cellular function [35]. We therefore set out to screen this list for proteins with developmental roles by systematically depleting them from zebrafish embryos using antisense morpholino oligonucleotides. This model system enables us to rapidly work through the centrosome inventory in a vertebrate host with fast and experimentally accessible tissue and organ development. Here, we analyse two genes involved in centrosome function. The absence of either gene in zebrafish results in 'ciliary' phenotypes, which closely resemble those of zebrafish mutants for intraflagellar transport genes (IFTs) [36,37].

## Results

**Figure 1:**
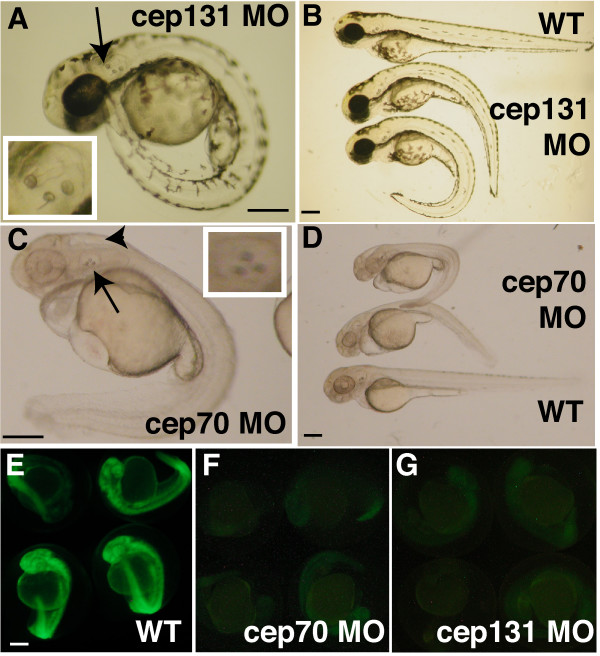
**cep70 and cep131 morphants at 48 h.p.f. **cep131 (A, B) and cep70 (C, D) with WT embryos shown at the top (B) and bottom (D) with morphants of varying severity as judged by curvature of the spine. The ectopic third otolith can be easily seen in (A) and (C), is labelled with an arrow and the otic vesicle is magnified in the insets. There is an arrowhead to the hydrocephaly present in the cep70 morphant in (C). To demonstrate morpholino effectiveness, embryos were co-injected with morpholino and an mRNA encoding the morpholino target site spliced in frame to GFP. In these embryos (F, G), expression was greatly reduced compared to embryos not injected with morpholino to another target sequence (E). Very little signal – much of it autofluorescence – could be detected when exposure time was increased to 8" for morphants (F, G) compared to 1/8" for control (E); otherwise panels would be blank. cep70 morphants and corresponding control embryos were treated with PTU hence the lack of pigmentation. Scale bar: 250 μm.

We conducted a pilot screen for centrosome proteins with developmental roles, injecting zebrafish embryos with morpholinos directed against the mRNAs for thirty centrosomal proteins that have been identified, focussing primarily on those that had not been well characterised. We were able to identify seven morpholinos that, upon early injection, resulted in morphological phenotypes (CJW & WAH, unpublished data). Two morphants particularly caught our attention. Embryos injected with morpholinos against the zebrafish homologues of cep70 and cep131 [35] displayed a curved back, slightly shortened body axis and ectopic otoliths were frequently observed (21/45 in cep70, 21/30 in cep131, not observed in same number of WTs) at 48 hours post-fertilisation (h.p.f.) (Fig. 1A–D). Both morpholinos gave a robust and highly penetrant phenotype.

We initially tested the effectiveness of these morpholinos against the desired sequence by modifying GFP to include the morpholino target site 5' and in-frame to the coding sequence and co-injecting mRNA for this construct with the morpholino. As expected, GFP fluorescence in the embryos was practically eliminated, which was not the case if a control morpholino was injected (Fig. 1E–G).

**Figure 2:**
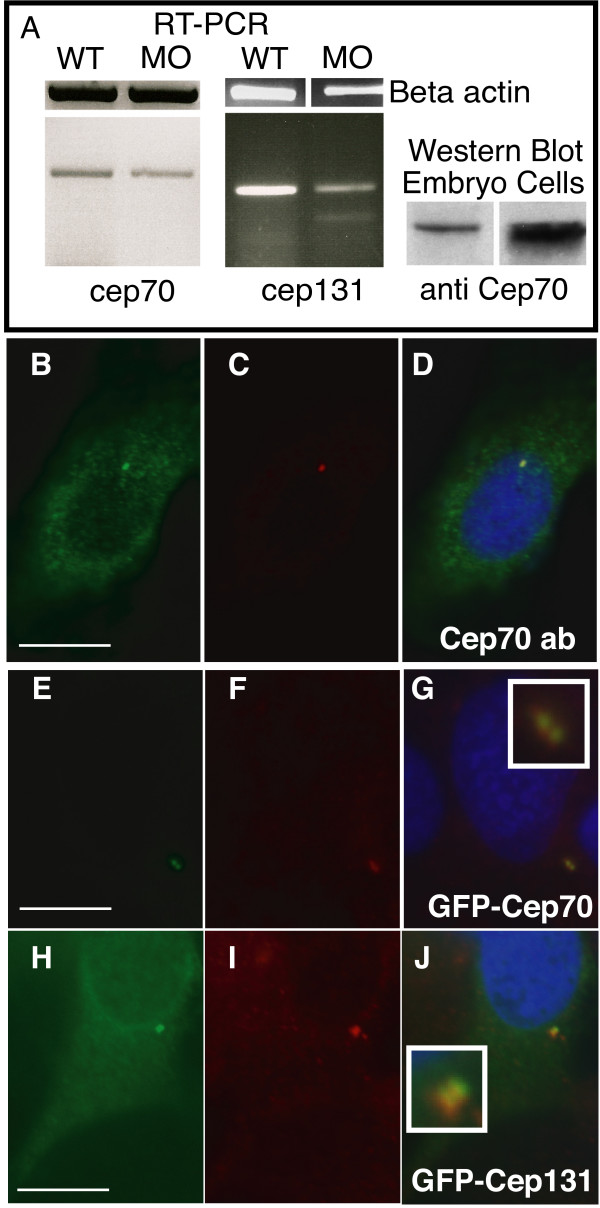
**Subcellular localisation of Cep70 and Cep131**. (A) Splicing-inhibitory morpholinos result in a reduction in cep70 and cep131 mRNA levels, as judged by RT-PCR of a 480 bp or a 360 bp fragment, respectively, that covers the splice site. In the cep131 morphant, an additional, faint band at 260 bp can be observed. This is consistent with skipping of the third exon, though other splicing products might also be possible. Right, an antibody to Cep70 recognises a 70 kDa band in PC2 cell line and zebrafish embryo extract. (B-J) Subcellular localisation of Cep70 and Cep131. An antibody raised to Cep70 gives a punctate staining (B) similar to gamma tubulin (C) in PC2 zebrafish cultured fibroblasts. The two signals coincide when the two channels are merged, the centrosome now shown in yellow (D). GFP-Cep70 and GFP-Cep131 localise to the centrosome, as expected (E-J). Cep70 or GFP fusions are shown in green, gamma tubulin is in red, nuclei are in blue (DAPI) in merged pictures. Insets enlarge the centrosome signal. Scale bar: 10 μm.

To show the specificity of the effect of depleting the proteins, we next tried to use splicing inhibitory morpholinos for both genes. Morpholinos were designed that bound the intron 3 – exon 4 junction of the predicted pre-mRNA of each gene. In both cases, a very similar phenotype of curved back and ectopic otoliths (cep70, 7/37; cep131, 12/30) was observed, although it was less severe for cep70 compared to the translation-inhibiting morpholino (data not shown). For each gene, then, two different morpholinos operating by different mechanisms – translation inhibition and inhibition of splicing – gave a very similar morphant phenotype. We used RT-PCR to assay if the level of mRNA of each gene had been reduced. In both cases, significant reduction was observed, though this was greater in the case of cep131 than cep70, as might be expected from the phenotype (Fig. 2A). We used the translation-inhibiting morpholinos in subsequent experiments as the severity of the phenotype suggests a greater level of depletion.

We cloned both cep70 and cep131 by RT-PCR from 24 h.p.f. zebrafish embryos and raised antibodies to recombinant zebrafish Cep70 overexpressed in E. coli and affinity purified. The recombinant protein was recognised by sera from rabbits immunised with zebrafish Cep70; one serum was used for subsequent experiments (rabbit anti-Cep70, polyclonal 485-4).

In both zebrafish PC2 cells and two-day old zebrafish embryo extracts, a band at the expected size could be detected by the rabbit anti-Cep70 serum, as well as a low molecular weight band in the cell line extracts. This protein was difficult to detect in embryos by Western blotting (Fig. 2A).

Immunofluorescence microscopy of PC2 cells labelled with anti-Cep70 antibody revealed a punctate, perinuclear pattern that coincided with that of gamma tubulin (Fig. 2B–D) and was absent from cells probed with the pre-immune serum. As expected, GFP-Cep70 and GFP-Cep131 localised to the centrosome (Fig. 2E–J). Two dots of signal were observed (Fig. 2G, J insets), strongly suggesting that these are present on or around both centrioles and not localised to one specific centriole or mother centriole-specific structures like the (sub-)distal appendages.

**Figure 3:**
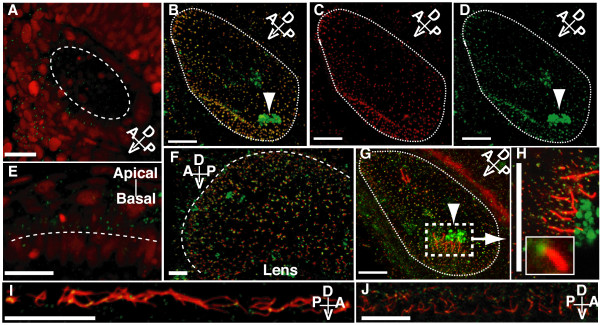
**Confocal wholemount (immuno)fluorescence localisation of Cep70 in zebrafish embryos**. (A and E) In embryos injected with GFP-Cep70 and H2B-RFP, the Cep70 signal, though weak, is observed at the apical surface of cells in the otic vesicle (A) and eye (E) at 24 h.p.f. . Long dashed lines indicate the apical surface: interior to the circle in (A) and above the line in (E). Other panels: 24 h.p.f. zebrafish embryos were probed with anti-Cep70 (green) and either anti-acetylated or anti-gamma tubulin (red). (B, C, D) Otic vesicle stained for gamma tubulin (C), Cep70 (D) and merged picture (B) showing that the two signals coincide. Arrowheads point to the non-specific staining of the otoliths. (F) and (G) show the eye and otic vesicle; Cep70 can be seen at the base of cilia stained for acetylated-tubulin. (H) shows the region around one of the clusters of tether cells in the otic vesicle where the position of Cep70 can be seen more clearly; an inset shows one basal body and cilium. Yellow colour, the overlap of Cep70 and acetylated-tubulin signal is due to the angle at which some of the cilia are seen. Again, a long dashed line indicates the apical surface of the eye in (F). Short dashed lines delineate the otic vesicle in (B, C, D and G). (I) and (J) show Cep70 and cilia in the pronephros and spine canal. Again the Cep70 can be seen at the base of cilia. Orientation of the specimens with regard to the embryo is shown by the compass in the panel: A = anterior, P = posterior, D = dorsal, V = ventral. Scale bar: 20 μm.

In 24 h.p.f. embryos, GFP-Cep70 localises to the apical surface of cells in the otic vesicle (Fig. 3A) and eye (Fig. 3E) with a punctate appearance. This can be replicated with wholemount immunofluorescence staining of 24 h.p.f. embryos with rabbit anti-Cep70 485-4 (Fig. 3F, G). This staining coincides with that of mouse monoclonal GTU-88 to gamma tubulin which labels centrosomes and basal bodies (Fig. 3B – merge of C and D) and lies at the base of cilia labelled with mouse monoclonal 6-11B-1 to acetylated tubulin (Fig. 3G), shown in detail in Fig. 3H and inset. In the otic vesicle there is non-specific staining of the otoliths but in other ciliated tissues, only the basal bodies are labelled (Fig. 3F (eye), I (pronephros), J (spine canal)). In ciliated cells, therefore, Cep70 localises to the basal bodies of cilia.

The morphological phenotype of the cep70 and cep131 morphants is similar to the zebrafish *oval *and *hippi *mutants, in which the zebrafish homologues of IFT88/Tg737^orpk^/Polaris and IFT57 are disrupted [36,37]. It has been found that defective cilia formation is the main cause for the phenotype of these embryos with altered IFT function. We reasoned that the cellular cause of the phenotype we observed should also lie in the state of embryonic cilia rather than the centrosomes themselves.

**Figure 4:**
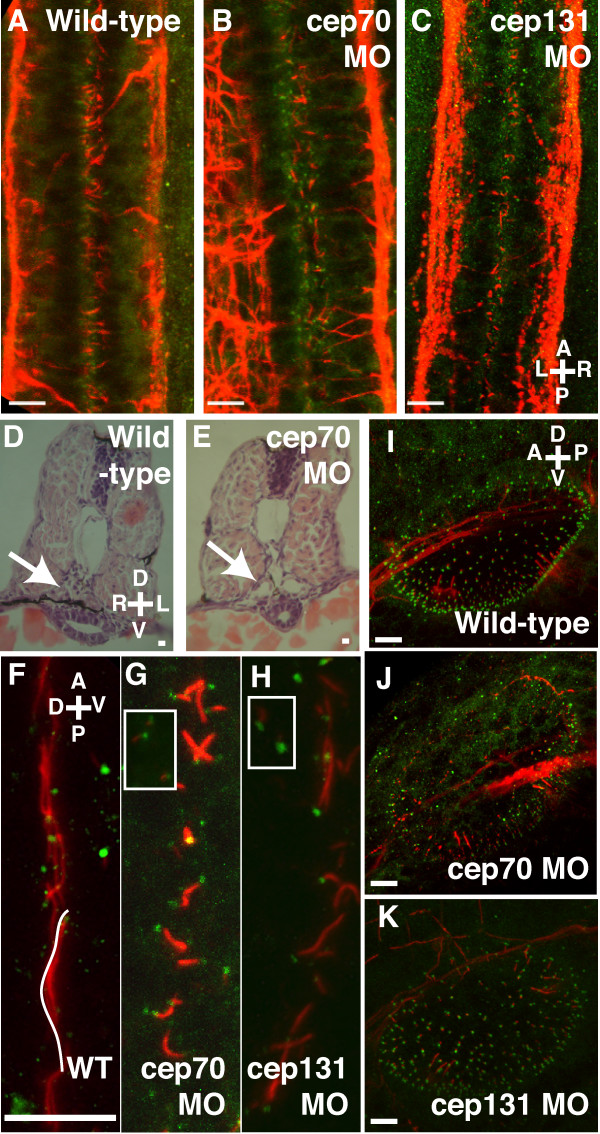
**Cilia in the otic vesicle, spine and pronephros at 30 h.p.f. **(A-C) Spine of WT, cep70 and cep131 morphants, stained for acetylated tubulin (red, cilia) and gamma tubulin (green, basal bodies). The cilia of the central canal can be seen in between the two nerve tracts. Cilia are shortened in the morphant embryos but basal bodies remain intact. Pronephric ducts – arrows – are enlarged in morphant cep70 embryos (E) compared to WT (D) at 48 h.p.f. . (F-H) Cilia are also reduced in length in the pronephros of the morphants (G, H) compared to WT (F). However, like other tissues, basal bodies are present. Basal bodies are also present in surrounding tissues (inset). One WT cilium has been colored white for clarity. (I-K) Otic vesicles of WT, cep70 and cep131 morphants. Compass in the panels shows orientation: A = anterior, P = posterior, D = dorsal, V = ventral, L = left, R = right. Scale bars: 10 μm.

We used confocal microscopy to analyse 30 h.p.f. morphant embryos, which were labelled for both gamma tubulin and acetylated tubulin. These mark centrosomes or basal bodies (γ-tubulin) and stabilised microtubules found in the axonemes of cilia (acetylated tubulin). We examined the cilia in the spinal canal, pronephros and otic vesicle. Motile cilia in the central canal of the spinal cord and pump fluid down this tube [36]. In the cep70 and cep131 morphants, these cilia are reduced in size from 3.5 ± 0.7 μm (n = 24) to 1.4 ± 0.6 μm (n = 37) and 1.7 ± 0.8 (n = 49) respectively (mean ± s.d.; Fig. 4A–C).

In zebrafish, motile cilia drive fluid flow down the tubules of the pronephros. In some IFT morphants, the pronephros expands to form a visible bubble at the head of the pronephros and histological sections of these embryos reveal much expanded pronephric ducts and tubule [36]. We did not observe such a severe phenotype although paraffin sections of cep70 morphants embryos showed a significant expansion in the ducts, which can also be observed in the confocal images of the ducts at an earlier stage (Fig. 4D, E and data not shown). This morphant phenotype is similar to that found in *oval *mutants, in which the pronephric cysts are mild compared to other mutants [36]. Analysis of the cilia in the pronephric ducts by confocal microscopy revealed a reduction in the length of the cilia from 9.0 ± 1.8 μm (n = 47) to 2.8 ± 1.4 μm in cep70 (n = 67) and 3.3 ± 1.6 μm (n = 39) in cep131 morphants (mean ± s.d.; Fig. 4F–H). However, basal bodies are still present in these cells, beneath the shortened cilia, so the effect on cilia is not due to the absence of centrioles and basal bodies from which the cilia extend.

The otic vesicles in zebrafish contain two types of cilia: two sets of long, tether cilia (5 μm) are surrounded by short, motile cilia about 1 μm long [38]. The former anchor the two otoliths normally observed in zebrafish embryos at this stage. The latter move the vesicle fluid that contains the calcium-protein particles that aggregate to form the otoliths. In the absence of effective cilia, these aggregates can form ectopically, rather than on the tether cilia [38]. The motile cilia themselves are too short to accurately compare the length between morphant and wild-type by immunofluorescence microscopy. By visual inspection, the size of the tether cilia, much longer than the motile cilia in the rest of the vesicle, is not obviously affected (Fig. 4I–K). In both morphants, basal bodies are still present in the ciliated cells and centrosomes in the surrounding tissue. Similar observations have been made with the *oval *mutant, in which ectopic otoliths are observed but the cilia of the otic vesicle appear normal [37] whereas other cilia – spine, pronephros – are shortened [36].

**Figure 5:**
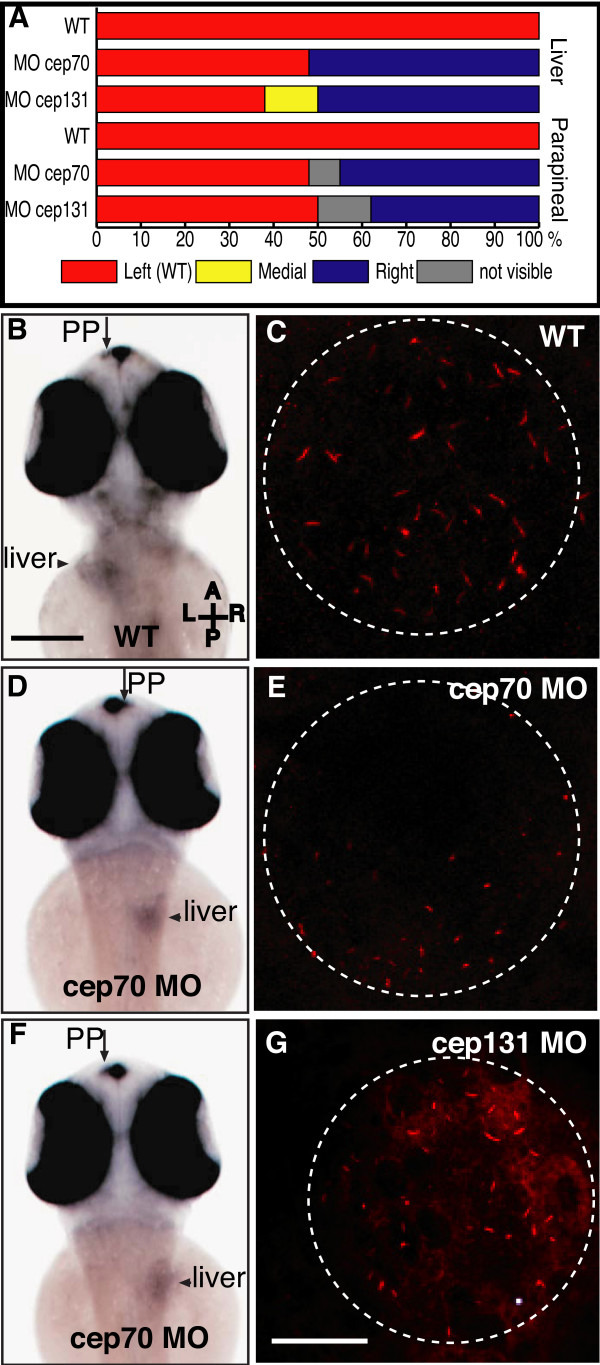
**Asymmetry in cep70 and cep131 morphants**. (A) Randomisation of asymmetry of liver and parapineal in cep70 (n = 27) cep131 (n = 16) compared to WT (n = 21) where both are on the left hand side. (B, D, F) Position of asymmetrically placed organs in cep70 morphants compared to WT, at 60 h.p.f., assayed by wholemount in situ hybridisation for *otx5 *and *fkd2 *which label the parapineal (PP) and liver. In WT (B), both are placed on the left hand side. In cep70 morphants, this is randomised but not concordantly: the embryo in (D) has the position of both liver and parapineal inverted whereas the embryo in (F) has them on opposite sides – liver inverted, parapineal normal. Scale bar: 250 μm. Compass shows embryo orientation in which A = anterior, P = posterior, L = left and R = right. (C, E and G) Cilia in Kupffer's vesicle in WT, cep70 and cep131 morphants. Cilia are much shorter in the morphant embryos. Dashed circle outlines the vesicle. Scale bar: 20 μm.

We next asked if other processes in which cilia are involved were also affected in these morphants. The Kupffer's vesicle (KV) is the zebrafish equivalent of the node in mouse embryos and harbours motile cilia, which are crucially involved in determining left-right laterality in both brain and body. The absence of these cilia or their lack of motility results in left and right in the developing embryos being randomly assigned [22,23,36]. Organs and tissues that are asymmetrically placed, such as the heart, liver, pancreas or parapineal, are on opposite sides to wild-type embryos (n = 21) in approximately half of the cep70 (n = 27) and cep131 (n = 16) morphant embryos affected (Fig. 5A), as revealed by wholemount in situ hybridisation with RNA probes to *otx5 *and *fkd2 *[39,40] (cep70 morphant, Fig. 5B, D, F). The placement of liver and parapineal is not established concordantly (compare Fig. 5D, both on opposite side, and Fig. 5F, liver on opposite side). We examined the KV by confocal microscopy, revealing no obvious changes in size and shape in cep70 morphants; cep131 morphant KVs were smaller (Fig. 5C, E, G). However, the cilia in the vesicles of both morphants are again much shorter, vestigial cilia of 1.5 μm (cep70 – mean 1.3 μm ± 0.5 s.d., n = 135; cep131 – mean 1.5 μm ± 0.6 s.d., n = 89) compared to 2–4 μm (WT mean 3.0 μm ± 0.9 s.d., n = 117) for those in normal embryos.

**Figure 6:**
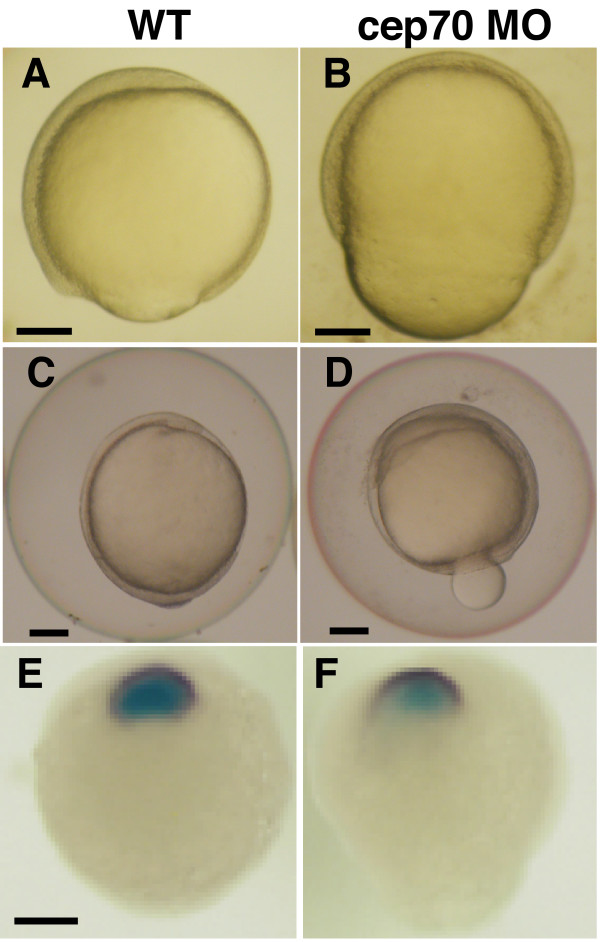
**Convergence-extension is unaffected in cep70 morphants**. (A-F) Defective epiboly in cep70 morphant (cep131 morphants normal at this stage). At 70% epiboly, morphant embryos (B) have an acorn shape, unlike normal development at this stage (A). As epiboly completes, morphant embryos (D) (WT shown in C) extrude some of the yolk but will recover and survive. Assaying convergence-extension by the distance between anterior neural margin and prechordal plate by wholemount in situ hybridisation for *dlx3 *(purple) and *hgg1 *(light blue) reveals no difference between morphant (F) and WT (E). Scale bar: 125 μm.

Defects in convergence and extension have been shown for some cilium morphants, such as the BBS proteins [41,42]. cep70 morphants have a phenotype similar to some mutants, such as silberblick and trilobite, whose role in convergence-extension are well established [43-45]. At the end of epiboly, cep70 morphant embryos adopt an acorn shape and part of the yolk is squeezed out as the germ ring contracts (Fig. 6A–D); their body axis is also shorter (Fig. 1). We assayed the effectiveness of convergence and extension in cep70 embryos by wholemount in situ hybridisation of tailbud stage embryos with RNA probes to *dlx3 *and *hgg1 *[46,47], which label the anterior neural margin and prechordal plate. In convergence-extension mutants, there is a significant gap between the two, which is absent in wild-type embryos (Fig. 6E) [44]. Such a gap is also absent in cep70 morphants (Fig. 6F) so, by this assay, early convergence-extension is proceeding normally.

**Figure 7:**
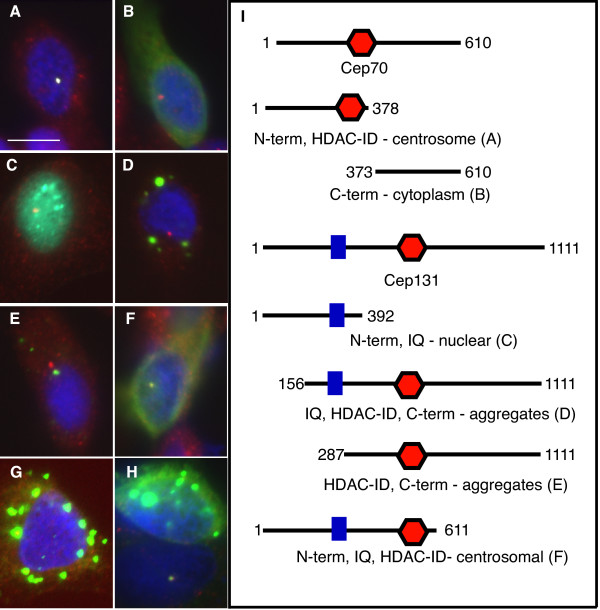
**Subcellular localisation of Cep70 and Cep131 fragments**. The N-terminus of Cep70 localises to the centrosome (A) whereas the C-terminus does not (B). Both the N-terminus, IQ (blue square) and HDAC-ID (red hexagon) domains of Cep131 are required for centrosomal localisation (F). (I) depicts which fragments were fused to GFP and the panels in which they are represented. (G) and (H) show aggregates generated by overexpression GFP fusions of the N-terminal fragment and full-length Cep70. GFP fusions are shown in green, gamma tubulin is in red, nuclei are in blue (DAPI) in merged pictures. Scale bar: 10 μm.

To investigate the cellular function of Cep70 and Cep131 in more detail, we analysed the peptide sequence of both proteins. Both Cep70 and Cep131 are coiled-coil proteins proposed by the SMART protein analysis tool to have a histone deacetylase-interacting domain (HDAC-ID) located in the middle of the peptide chain. HDAC6 is, in fact, also a tubulin deacetylase [48] so these domains could be relevant to the function of these proteins in ciliogenesis. Cep131 also possesses an IQ domain in its N-terminus. To assess whether these domains are responsible for centrosomal association or function of these proteins, we expressed both Cep70 and Cep131 as fragments in PC2 cells. The HDAC-ID-containing half of Cep70 did indeed localise to the centrosome (Fig. 7A) whereas the other half of the protein only showed a cytoplasmic staining (Fig. 7B). The behaviour of the Cep131 fragments is more complex. On its own, the N-terminal third minus the HDAC-ID displays nuclear localisation (Fig. 7C); only a fragment containing both the native N-terminus, IQ and HDAC-ID domains shows centrosomal localisation (Fig. 7F). Other fragments form cytoplasmic aggregrates that do not always co-localise with the centrosome (Fig. 7D, E). None of these fragments had any notable effects on the cells, apart from at high expression levels where the large aggregates appeared to be toxic.

We tried to rescue the cep70 morphant by co-injecting, sequentially, the morpholino against cep70 and an mRNA encoding the N-terminal half of Cep70. This protein fragment, when injected at moderate to high amounts (> 100 ng), resulted in severe defects and toxicity and did not rescue the morphant, presumably reflecting the effect of the aggregates that can form in transfected cells (GFP-fusions of full-length Cep70 and N-terminal fragment, Fig. 7H, G).

To test for interaction between these proteins we used the yeast two-hybrid assay with these combinations: Cep70 against Cep131; either Cep70 or Cep131 against HDAC6; and either Cep70 or Cep131 versus members of the IFT family of proteins (IFTs 52, 57, 88). We could not detect any interaction between Cep70 or Cep131 and HDAC6, IFT52, IFT57 or IFT88. Cep70 and Cep131 did not interact with each other by this assay but Cep70 did bind itself (data not shown). As it is a predicted coiled-coil protein, this is not surprising.

**Figure 8:**
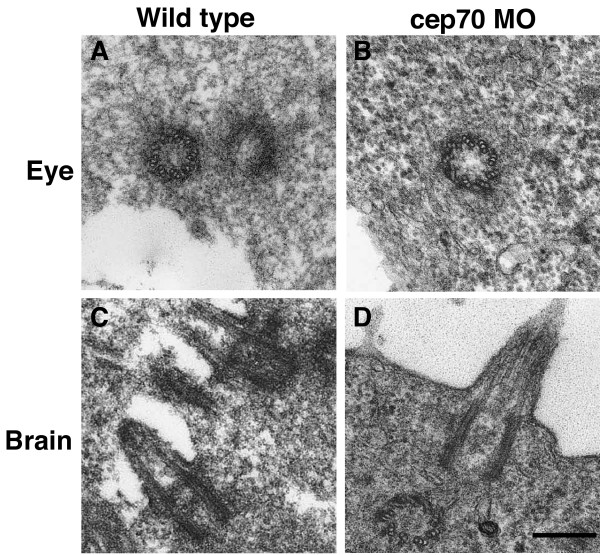
**Micrographs of basal bodies and cilia from WT and cep70 morphant zebrafish embryos**. An eye centriole and brain cilium from 24 h.p.f. WT (A, C) and morphant (B, D) are shown. Scale bar: 250 nm.

Lastly, we looked at the ultrastructure of basal bodies and centrioles in cep70 morphant zebrafish embryos but could observe no difference to the wild-type organelles (Fig. 8A–D): all nine triplet microtubules of basal bodies or centrioles appeared intact and axonemes could be observed emerging from the basal bodies in the brain, where hydrocephaly is observed (Fig. 8A–D and Fig. 1C, arrowhead). Unlike some mutants with similar phenotypes (e.g. *fleer *where axonemal microtubules are disrupted [49]) there were no obvious, gross defects in the centrioles. Whilst we cannot exclude the possibility of fine ultrastructural changes in the tissues affected, centrioles and basal bodies are clearly present in the embryo so the ciliary-related defects are not due to a global absence of centrioles.

## Discussion

The gross anatomical phenotype of the morphants depleted for Cep70 or Cep131 – the extra otoliths and curved back – have been observed before in zebrafish morphants in intraflagellar transport proteins. In the case of IFT88 and IFT57 morphants, as for Cep70 and Cep131 morphants, cilia are shortened but not completely eliminated in Kupffer's vesicle, the spine and pronephros [36]. Cilia disappear at later stages (3 d.p.f.) in eye and ear in the oval mutant (IFT88) [37]. Centrosomes and basal bodies/centrioles are still present in Cep70 and Cep131 morphants so the organelle that seems to be most vulnerable to depletion of these proteins is the cilium rather than the basal body/centriole from which it grows. With the levels of depletion we achieve, although the cilia are much reduced in length, they are not completely eliminated. Indeed, similar to Vladar and Stearns [50], Graser and Nigg [51] have found that a number, but by no means all, centrosome proteins contribute to ciliogenesis of the primary cilium of cultured retinal pigmented epithelial cells. So far though, only one, Cep164, can be depleted with almost complete elimination of cilia but preservation of the basal body [51], similar to the 'end-point' of the oval mutant [37].

The motile cilia of the otic vesicle are already of a size to which other cilia in the cep70/131 and IFT57/88 morphants are reduced. This might make them resistant to defects in the ciliogenesis pathway in which Cep70 and Cep131 are involved that reduce the length of other cilia. Any proportional changes in length would also not be accurately measurable with our techniques. The motility of the cilia could still be impaired, which would also result in the formation of the ectopic otoliths observed. The tether cilia appear normal whereas, based on the pronephric cilia, one would expect a clear, visible reduction in length to that of the neighbouring motile cilia. Subtle differences between cilia and cell types in different tissues could render these proteins redundant in certain cells. It is not the case that depletion of any (peri)centriolar protein has the same effect on all cilia in the zebrafish embryo. Depletion of IFT proteins in zebrafish affects nearly all cilia [36,37]. Zebrafish morphants for the Bardet-Biedl syndrome genes show normal eye development, do not exhibit a curved back or ectopic otoliths but do display randomisation of left-right asymmetry [42]. Morphants of the Joubert syndrome/nephronophthisis-associated protein Cep290 have certain defects with cerebellum morphology and have kidney cysts but both length and movement of the pronephric cilia is unaffected [52]. Indeed, Zhao and Malicki have recently identified a set of zebrafish mutants in which subsets of cilia are affected although the subcellular localisation of the gene products has yet to be determined [53].

Morphants for zebrafish IFT78 and IFT88 homologues closely but not completely resemble the two morphants described here, both at the gross anatomical, cellular and subcellular level [36]. IFT57/88 morphants show reduced cilia length in the KV, pronephros and spine central canal as we observe in the two Cep70/131 morphants. The IFT family of proteins might therefore contain candidate partner proteins. However, we could not detect an interaction between Cep70 or Cep131 and several of the IFT proteins. Our search was limited, though, and it remains possible that, as more of these proteins are identified, one member of the IFT family could still be the partner for these two proteins.

Other proteins that could be partners of both Cep70 and Cep131 are the histone deacetylases as both Ceps possess HDAC interacting domains. However, the exact role of these domains is not immediately obvious. The HDAC that deacetylates tubulin reverses ciliogenesis [54]. Unless these Ceps sequester HDAC6 at the basal body, localising active HDAC6 to cilia via Cep70 and Cep131 would prevent ciliogenesis and so depleting these proteins would prevent cilium reabsorbtion or deciliation. Whilst the HDAC-ID of Cep70 is in the centrosome-localising region of this protein, this domain is not the sole determinant of centrosomal localisation in Cep131, which would also suggest that these proteins would act as anchors for the enzyme, not the other way around. Again, though, our assays failed to detect an interaction between these proteins. This could be a technical limitation or the HDAC-ID, in fact, anchors another HDAC or another molecule.

## Conclusion

We have described here a role for previously uncharacterised centrosome proteins, Cep70 and Cep131, as contributors to ciliogenesis in certain tissues in the developing vertebrate (zebrafish) embryo. It is as components of the basal bodies of cilia rather than centrosome proteins per se that these proteins appear to exert their effect in these morphants. Neither protein interacts with several IFTs or the deacetylase HDAC6, which are known to have key roles in extending and maintaining cilia, so, if Cep70 and Cep131 are involved in these pathways, other proteins probably act as interlocutors. Alternatively, these two proteins act in another pathway that controls cilium length. The resemblance of both cep70 and cep131 morphants to those of zebrafish IFT57 and IFT88 makes the two proteins candidates for genes mutated in inherited human diseases similar to Bardet-Biedl syndrome in which cilia in a number of tissues are affected. Further investigation of their function should yield valuable knowledge on the function of cilia and their roles in embryonic development and disease.

## Methods

### Zebrafish

Zebrafish were maintained and bred at 26.5°C; embryos were raised at 28.5°C, as described by Westerfield [55]. Both AB and TL wild-type strains were used for these studies. For some studies, embryos were grown in 0.003% phenylthiourea to inhibit melanin production. Embryos were injected in the yolk with morpholinos or mRNA using a micromanipulator-mounted micropipette (Borosil 1.0 × 0.5 mm, Frederick Haer & Co., Inc., USA) and a Picospritzer microinjector. Between 1–5 nl of solution were injected into the yolk of embryos. Morpholinos directed against cep70 and cep131 were purchased from Gene Tools, Inc., USA via Openbiosystems, Inc., USA: MOzcep70st, 5' TTCTCTCTGTTCTTCCTGCTCCATC 3'; MOzcep131st, 5' ATGGACTGCGGGTTGTATGCATCTT 3'. They were used at 1 ng and 2 ng, respectively, injected at the 1–4 cell stage. Anti-splicing morpholinos were injected at 0.6 and 0.4 pmol respectively for MOcep70i3e4 5' GCACTTCCTCTCTGGAAATAAACAA 3' and MOcep131i3e4 5' TCAGTCTGCCAACAACAGAGAGTGC 3'. 100–200 pg of mRNA was injected; synthesis is described below.

### Histology

Zebrafish embryos were routinely fixed in 4% paraformaldehyde in phosphate-buffered saline (PBS). Cryosections were made at 10 μm thickness from OCT-mounted zebrafish embryos using a Leica 2800E cryostat and mounted on SuperFrost Plus slides (Menzel, Germany). OCT was removed with several washes of PBS before processing for immunofluoresence. Paraffin sections were made at 5 μm using a Leica microtome. Embryos were embedded in paraplast paraffin (Sigma, St Louis, USA) through a series of water to ethanol to Histoclear™ substitutions. Sections were stained with haematoxylin and eosin and coverslips mounted with DPX (Fluka/Sigma-Aldrich).

### Immunofluorescence microscopy

Both slides of cryostat-sectioned embryos and coverslips on which cells had been cultured were blocked before incubation with antibodies for 30 minutes at room temperature in 10% heat-inactivated goat serum, 1% bovine serum albumin, 0.2% Triton X-100 in PBS. Primary and secondary antibodies were incubated for 1 hour at room temperature in blocking solution. Between and after incubations, samples were washed with PBS. Slides and coverslips were mounted in Fluorsave (Calbiochem, USA) or DABCO in 90% glycerol and PBS.

The primary antibodies were: rabbit anti-gamma tubulin (Sigma, cat. no. 5192), 0.6 μg/ml; mouse anti-acetylated tubulin 6-11B-1 (Zymed, USA), 1 μg/ml; mouse anti-rhodopsin RET-P1 (Abcam, UK), 2 μg/ml; mouse anti-gamma tubulin, monoclonal GTU-88 (Sigma, USA), 1 μg/ml. Secondary antibodies used were: goat anti-mouse IgG Cy3-conjugated (Chemicon, Temecula, CA, USA), 1 μg/ml; goat anti-mouse IgG and goat anti-rabbit IgG Alexa 488-conjugated (Molecular Probes, Eugene, OR, USA), 2 μg/ml. Nuclei were counterstained with DAPI (4',6-diamidino-2-phenylindole) at 0.1 μg/ml.

For whole-mount immunostaining, embryos were permeabilised by incubating the embryos in 0.25% trypsin-EDTA in Hanks Balanced Salt Solution (Gibco, USA) for 10 min on ice then washed three times for 30 min in PBS plus 0.2% Triton to remove the enzyme. Blocking was extended to 4 hours, antibody incubations to 36 hours at 4°C and antibody dilutions halved.

Photomicrography was performed with either Leica TCS SP series laser confocal systems or with Nikon upright fluorescence microscopes, equipped with cooled charge-coupled device (CCD) Hamamatsu Orca cameras controlled through Openlab software (Improvision, USA). Confocal stacks were analysed using Volocity image analysis software (Improvision, USA). Stacks were taken in 0.5 μm or 1 μm sections and are represented as maximum intensity projections of all of the stack. Cilia lengths from wild-type and morphant embryos were found to be significantly different by paired T-tests with P values of 0 at 3 decimal places. Variations on the statistical model, including analysis of variance models gave the same result.

### Molecular cloning

Molecular cloning followed standard protocols [56] and the instructions of the manufacturer of the kits, reagents and enzymes used. All restriction enzymes were obtained from New England Biolabs and polymerases from Stratagene. cep70 and cep131 were cloned by RT-PCR from 24 h.p.f. zebrafish embryos using the Machery-Nagel NucleoSpin RNA II kit to obtain total RNA, MuLV reverse transcriptase and QTaq thermostable DNA polymerase (both from BD Biosciences, USA) were used to generate cDNA and then amplify specific sequences. polyT20V oligonucleotide was used to prime mRNAs for reverse transcription. The following oligonucleotides were then used to amplify and modify cep70 and cep131 cDNAs: oCJW28.1 TTTGGATCCTGATGGAGCAGGAAGAACAGAGAG; oCJW28.2 TTTCTCGAGGCCTTTGAGGGAACGGATTCTTGG; oCJW29.1 TTTGGATCCTCATGCATACAACCCGCAGTCCATCTG; oCJW29.8 TTCTCGAGTTTCCCTAACAGCTGTTTTCTCTGC. cDNAs were first cloned in pGEM-T'easy (Promega, USA). The ends of the coding sequences of these genes were altered by PCR to enable them to be cloned in-frame into pCS2P+EGFPN [57]. Both cep70 (oCJW28.1 & oCJW28.2) and cep131 (oCJW29.1 & oCJW29.8) were altered to have BamH I and Xho I sites at their 5' and 3' ends respectively so they could be inserted into the Bgl II and Sal I sites of pCS2P+EGFPN. mRNAs were transcribed from the Sp6 promoter of this vector using the mMessage mMachine in vitro transcription kit (Ambion, TX, USA). RNA was purified using the Qiagen RNeasy kit (Qiagen GmbH, Germany). Natural restriction sites in cep70 and cep131 were used to splice fragments of these genes to GFP in pEGFP-C1/2 (Clontech) for overexpression in zebrafish PC2 cells.

Zebrafish HDAC6, IFT52, IFT57 and IFT88 were cloned similarly to the above using these oligonucleotide primers: TTTGATCACGATGGATGCGGTTCCAGATACC & TTCTCGAGGTTGAAAGGATGAATCCCCTCTCC (HDAC6, Bcl I & Xho I sites); TTAGATCTCCATGGACAAAGAGCAAAGAAATATCG & TTGTCGACTCAGTACATGCTGAACCTCGCTTC (IFT52, Bgl II & Sal I); TTTAGATCTCCATGGCGGAGGAGGAAGAGCGCGG & TTTGTCGACATAAGCCTGCGCATTGGGCTCTAAG (IFT57, Bgl II & Sal I); TTTGATCACGATGGAGAATGTGCATCTTGTCCC & TTGTCGACCTCAGGCAGTAAATCATCTCCCAG (IFT88, Bcl I & Sal I). The coding regions of these genes were then inserted into yeast two-hybrid vectors pACT2 and pGBT9 at the BamH I and Xho I sites of these vectors. RT-PCR was performed using the Qiagen micro-RNA preparation and one-step RT-PCR kits, according to manufacturer's instructions. Two pairs of primers – AGTTGGTTTTGTTGGAGCGAAAG & TGGTCATTGGTGGAGTTGGGTC and CGCAGTCCATCCGCATCCATCC & TGGACATCTTGGAGAGGCTGGC – were used to amplify a region of cep70 and cep131, respectively, to test the efficacy of the splicing-inhibitory morpholinos.

### Wholemount in situ hybridisation

Digoxygenin (DIG)-labelled RNA probes were transcribed in vitro from *otx5*-, *fkd2*-, *hgg1*- and *dlx3*- containing plasmids using Roche T7 and T3 RNA polymerases. Embryos were refixed in methanol at -20°C overnight then rehydrated in PBS plus 0.1% Tween-20. After substituting the PBS with HYB (50% formamide, 5× SSC pH 6, 0.1% Triton X-100), embryos were incubated for 4 h at 65°C, then the probes were added in HYB plus torula RNA (50 μg/ml) and heparin (50 μg/ml) and the incubation continued for 36 h. The embryos were washed with HYB which was substituted with PBS Tween through 2× and 0.2 × SSC (1 × SSC = 150 mM sodium chloride, 15 mM sodium citrate). Embryos were incubated with the blocking solution described above for 4 h then anti-digoxygenin antibody coupled to alkaline phosphatase (GE Healthcare, UK) was added and the incubation continued overnight at 4°C for 24 h After extensive washing in PBS overnight, the embryos were developed in BM Purple (Roche, Germany). Ratios of left:right for position of liver and parapineal were significant by Fisher's Exact Test with P values of less than 0.01.

### Cell culture

PC2 cells (generated by P. Culp in the laboratory of N. Hopkins [58]) were a kind gift of Wolfgang Driever, with the permission of Nancy Hopkins. Cells were grown in Corning 25 and 75 cm^2 ^vent-capped flasks and 6-well plates (Nunc, Denmark) in DMEM:F12 supplemented with 10% foetal bovine/calf serum and 1% antibiotic mix (all from Gibco/Invitrogen) at 29°C and 6% CO_2_. The doubling time of the cells was about 48 h. Ethanol-washed coverslips were added to the 6-well plates to enable subsequent processing for immunofluorescence microscopy. These coverslips were fixed in methanol at -20°C before antibody incubation.

### Anti-Cep70 antibody

cep70 was subcloned in pET28b(+) and the protein overexpressed in E. coli as an insoluble His-tagged protein. This protein was purified as described by Sambrook and Russell (2001) using Ni-NTA resin (Invitrogen) following the instructions of the manufacturer. Rabbit antibodies to Cep70 were raised in New Zealand white rabbits by Charles River Laboratories (Germany).

### SDS-PAGE and Western blotting

Cell and embryo extracts were analysed by SDS polyacrylamide gel electrophoresis followed by Western blotting as described by Sambrook and Russell (2001) and Harlow and Lane [59] using the Mini-Protean gel apparatus and transfer cell from Bio-Rad (USA). Nitrocellulose blots (GE Healthcare/Amersham, UK) were stained with 1% Ponceau to demarcate the lanes; prestained markers also from Bio-Rad were used as molecular weight standards. After rinsing with PBS + 1% Tween-20, blots were blocked in PBS + 5% skimmed milk powder + 0.1% Tween-20 for 1 h at RT. Primary and secondary antibody incubations used the same blocking solution and incubation conditions. After each antibody incubation, blots were rinsed once in PBS-Tween and washed twice for 15 min in the same solution. Antibodies used were: rabbit anti-Cep70, polyclonal 485-4, serum at 1:500 dilution; rabbit anti-GST, polyclonal, A5800 (Molecular Probes), 1 μg/ml; mouse anti-gamma tubulin, monoclonal GTU-88, 1 μg/ml; goat anti-rabbit IgG, 0.15 μg/ml (Zymed, USA); goat anti-mouse IgG, HRP conjugate, 1 μg/ml (Abcam, UK).

### Yeast two-hybrid assays

Yeast strain AH109 (Clontech) was transformed with pACT2- and pGBT9- based constructs as described by Gietz and colleagues [60,61]. Double and single transformants were selected on solid yeast drop-out medium (Difco yeast nitrogen base plus Sigma Y1376 yeast synthetic drop-out medium supplements in ddH_2_O plus agar (Difco), as manufacturer's instructions) supplemented with histidine, uracil, leucine and tryptophan as appropriate. Transformants were initially selected for the presence of both plasmids then plated on triple-selective media to detect interactions between the two test proteins encoded on the pACT2-/pGBT9-plasmid constructs.

### Electron microscopy

30 h.p.f. zebrafish embryos were fixed by immersion in 2% glutaraldehyde containing 2 mmol/l CaCl_2 _in 0.1 M PIPES buffer at pH 7.4 with 100 μl 33% H_2_O_2 _added to each 10 ml aliquot immediately before use. After fixing in suspension for 2 hours a 4°C, they were rinsed twice in 0.1 M PIPES buffer pH 7.4 and transferred to 1.5 ml glass tubes. They were post-fixed in 1% osmium ferricyanide for 1 hour, rinsed 3 times in ddH_2_O and bulk stained in 2% uranyl acetate for 1 hour. They were rinsed in ddH_2_O and dehydrated in an ascending series of ethanol solutions to 100% ethanol, rinsed twice in acetonitrile and embedded in quetol epoxy resin. 50 nm sections were cut on a Leica Ultracut UCT, stained with saturated uranyl acetate in 50% ethanol and lead citrate and viewed in a FEI Philips CM100 operated at 80 kv.

## Abbreviations

ab: antibody; BBS: Bardet-Biedl syndrome; d.p.f: days post fertilisation; h.p.f.: hours post fertilisation; HDAC: histone deacetylase; IFT: intraflagellar transport; KV: Kupffer's vesicle; PKD: polycystic kidney disease.

## Authors' contributions

CJW devised project strategy, performed the experiments and analysis and wrote the manuscript. MC performed left-right asymmetry assays and analysis. WAH advised CJW on project strategy and aided with analysis and writing the manuscript. All authors read and approved the final version.
